# Prior intracerebral hemorrhage and white matter hyperintensity burden on recurrent stroke risk

**DOI:** 10.1038/s41598-021-96809-3

**Published:** 2021-08-31

**Authors:** Jong-Ho Park, Sun U. Kwon, Hyuk Sung Kwon, Sung Hyuk Heo

**Affiliations:** 1 Department of Neurology, Myongji Hospital, Hanyang University College of Medicine, Goyang, Korea; 2 Department of Neurology, Asan Medical Center, University of Ulsan College of Medicine, Seoul, Korea; 3 Department of Neurology, Hanyang University College of Medicine, Seoul, Korea; 4 Department of Neurology, Kyung Hee University Hospital, Seoul, Korea

**Keywords:** Neuroscience, Neurology

## Abstract

Prior intracerebral hemorrhage (ICH) is associated with increased risk of ischemic stroke. Since white matter hyperintensity (WMH) is associated with ischemic stroke and ICH, this study aimed to evaluate the relationship between ICH and the risk of recurrent stroke by WMH severity. From a prospective multicenter database comprising 1454 noncardioembolic stroke patients with cerebral small-vessel disease, patients were categorized by presence or absence of prior ICH and WMH severity: mild-moderate WMH (reference); advanced WMH; ICH with mild-moderate WMH; and ICH with advanced WMH. Among patients with ICH, the association with stroke outcomes by WMH burden was further assessed. The primary endpoint was ischemic stroke and hemorrhagic stroke. The secondary endpoint was major adverse cardiovascular events (MACE): stroke/coronary heart disease/vascular death. During the mean 1.9-year follow-up period, the ischemic stroke incidence rate per 100 person-years was 2.7, 4.0, 2.5, and 8.1 in increasing severity, and the rate of hemorrhagic stroke was 0.7, 1.3, 0.6, and 2.1, respectively. The risk of ischemic stroke was higher in ICH with advanced WMH (adjusted HR 2.62; 95% CI 1.22−5.60) than the reference group, while the risk of hemorrhagic stroke trended higher (3.75, 0.85–16.53). The risk of MACE showed a similar pattern in ICH with advanced WMH. Among ICH patients, compared with mild WMH, the risk of ischemic stroke trended to be higher in advanced WMH (HR 3.37; 95% CI 0.90‒12.61). Advanced WMH was independently associated with an increased risk of hemorrhagic stroke (HR 33.96; 95% CI 1.52−760.95). Given the fewer rate of hemorrhagic stroke, the risk of hemorrhagic stroke might not outweigh the benefits of antiplatelet therapy for secondary prevention.

## Introduction

Primary intracerebral hemorrhage (ICH) is an important public health issue as it leads to high rates of mortality and stroke-related disability in adults^1^. Stroke clinicians are faced with a therapeutic dilemma when treating ICH survivors who are at high risk of thrombotic events because antithrombotic medication used for secondary stroke prevention may increase the risk of recurrent ICH^2^.

Among cerebral small-vessel disease (cSVD) phenotypes, increasing severity of white matter hyperintensity (WMH) is associated with both ischemic and hemorrhagic stroke risk^3–6^. While cerebral microbleeds (CMBs) are associated with increased risk of subsequent ICH in individuals after ischemic stroke or transient ischemic attack^7^ and in ICH survivors^8^, there may also be a dose-dependent association between CMB burden and ischemic stroke risk^9^. A recent observational cohort study found that the risk of ischemic stroke after ICH is at least as high as that of recurrent ICH, the finding of which is much higher for patients after deep ICH^10^. Another population-based cohort study also found that risk of ischemic stroke increased sixfold in the first 6 months after ICH in elderly patients^11^.

Because ICH shares a similar pathophysiology with cSVD^12^, more information about the relationship of ICH and recurrent stroke risk (ischemic vs. hemorrhagic) according to WMH severity could be informative. This study aimed to evaluate associations between the presence or absence of ICH according to WMH severity among patients after noncardioemboloic stroke and subsequent stroke characteristics. Among ICH-carrying individuals, the association between WMH burden and stroke outcomes was also assessed.

## Results

### Baseline characteristics by cSVD phenotypes.

Of the 1,534 subjects in the PICASSO database, 80 subjects (5.2%) with no baseline MRI data were excluded, 1,454 subjects were analyzed in this study (62.2% male; mean age, 65.9 ± 10.8 years; mean follow-up period, 1.9 ± 1.3 years [median, 1.8; IQR, 1.0‒3.0]). The study subjects had a high burden of cSVD (CMB in 74.5% of patients with a median [IQR] of 3 [1–7] CMB; ICH in 38.8% of patients; and lacune in 83.4% of patients). Baseline demographic and clinical characteristics by cSVD phenotypes are provided in Table 1. When compared with the reference group, subjects with ICH with advanced WMH were older, had higher qualifying stroke severity, presence of lacune, number of CMB, and were more likely to have numerous CMBs (≥ 10). Subjects with ICH with advanced WMH were less likely to be male or to be smokers, and statin medication use, MMSE scores, and serum levels of uric acid were lower than in subjects with mild-moderate WMH.

**Table 1:** Baseline demographic and clinical characteristics by cSVD phenotypes among individuals after recent noncardioembolic stroke.

	cSVD phenotypes				P*
	Mild-moderate WMH (n = 638)	Advanced WMH (n = 252)	ICH with mild-moderate WMH (n = 440)	ICH with advanced WMH (n = 124)	
**Demographics**					
Age, years	65.1 ± 11.1†	69.4 ± 9.8‡	64.2 ± 10.7	69.0 ± 9.1‡	< 0.001
Male sex	448 (70.2)†	109 (43.3)‡	288 (65.5)	59 (47.6)‡	< 0.001
**Medical history**					
Hypertension	563 (88.2)	221 (87.7)	394 (89.5)	116 (93.5)	0.313
Diabetes mellitus	209 (32.8)	69 (27.4)	153 (34.8)	34 (27.4)	0.145
Dyslipidemia	291 (45.6)†	111 (44.0)	171 (38.9)‡	53 (42.7)	0.175
Coronary heart disease	33 (5.2)	9 (3.6)	21 (4.8)	4 (3.2)	0.649
Current smoking	328 (51.4)†	78 (31.0)‡	199 (45.2)‡	39 (31.5)‡	< 0.001
Family history of stroke	142 (22.3)	48 (19.0)	101 (23.0)	23 (18.5)	0.514
**Qualifying ischemic event**					0.668
Ischemic stroke	607 (95.1)	241 (95.6)	418 (95.0)	115 (92.7)	
Transient ischemic attack	31 (4.9)	11 (4.4)	22 (5.0)	9 (7.3)	
Time to randomization, days	18.0 (7.8, 36.0)	16.0 (7.0, 37.5)	18.0 (7.0, 41.8)	19,5 (12.0, 37.5)	0.713
Qualifying stroke NIHSS	1 (0, 3)†	2 (1, 3)‡	2 (1, 4)‡	2 (1, 4)‡	< 0.001
**MRI marker**					
Presence of lacune	517 (88.2)†	231 (98.3)‡	356 (87.3)	109 (97.3)‡	< 0.001
CMB, n	4.8 ± 7.3† (n = 586)	11.8 ± 18.2‡ (n = 235)	3.6 ± 8.1 (n = 408)	12.3 ± 21.7‡ (n = 112)	< 0.001
CMB, n 0 to 1 (n = 488)	183 (31.2)†	34 (14.5)‡	242 (59.3)‡	29 (25.9)	< 0.001
2 to 4 (n = 387)	220 (37.5)†	63 (26.8)‡	77 (18.9)‡	27 (24.1)‡	
5 to 9 (n = 240)	110 (18.8)†	60 (25.5)‡	51 (12.5)‡	19 (17.0)	
≥ 10 (n = 226)	73 (12.5)†	78 (33.2)‡	38 (9.3)	37 (33.0)‡	
**Vital signs**					
Systolic BP, mm Hg	136.5 ± 18.7	134.2 ± 17.5	135.1 ± 18.1	133.4 ± 16.6	0.169
Diastolic BP, mm Hg	80.8 ± 12.4	79.8 ± 11.4	80.2 ± 11.2	79.2 ± 10.9	0.440
Heart rate (beats per min)	81.7 ± 14.9	81.2 ± 14.3	79.3 ± 13.7	81.9 ± 13.0	0.044
Body mass index, kg/m^2^	24.4 ± 3.7	24.4 ± 3.7	24.5 ± 3.3	23.9 ± 3.5	0.546
MMSE, score	25.1 ± 4.7†	21.7 ± 6.3‡	24.1 ± 5.5‡	20.1 ± 6.8‡	< 0.001
**Laboratory findings**					
Glucose, mg/dL	121.2 ± 44.3	117.2 ± 45.8	121.6 ± 45.1	114.1 ± 36.4	0.278
HbA1c, %	6.2 ± 1.1†	6.1 ± 1.0	6.4 ± 1.4‡	6.0 ± 1.0	0.003
Total cholesterol, mg/dL	169.5 ± 41.1	167.5 ± 38.7	170.2 ± 43.5	165.4 ± 38.5	0.671
LDL-C, mg/dL	103.8 ± 36.0	102.0 ± 31.9	103.2 ± 37.6	102.7 ± 35.8	0.926
Triglycerides, mg/dL	135.7 ± 95.2	125.0 ± 84.7	127.2 ± 82.4	114.8 ± 61.5	0.073
HDL-C, mg/dL	45.1 ± 12.3	45.7 ± 11.4	45.0 ± 11.6	45.5 ± 12.5	0.868
Creatinine, mg/dL	1.01 ± 0.66	0.98 ± 0.45	0.99 ± 0.44	0.98 ± 0.67	0.811
Uric acid, mg/dL	5.4 ± 1.5†	5.0 ± 1.7‡	5.1 ± 1.7	4.8 ± 1.4‡	0.001
**Concomitant medication**					
Antihypertensive	465 (72.9)	183 (72.6)	323 (73.4)	98 (79.0)	0.537
Statin	538 (84.3)†	198 (78.6)‡	338 (76.8)‡	86 (69.4)‡	< 0.001
Probucol	328 (51.4)	127 (50.4)	222 (50.5)	55 (44.4)	0.558
**Antiplatelet drug**					0.462
Cilostazol	309 (48.4)	131 (52.0)	224 (50.9)	55 (44.4)	
Aspirin	329 (51.6)	121 (48.0)	216 (49.1)	69 (55.6)	

Values provided are number (%), mean ± SD, or median (interquartile range).

*cSVD* cerebral small-vessel disease, *WMH* white mater hyperintensity, *ICH* intracerebral hemorrhage, *NIHSS* National Institutes of Health Stroke Scale, *MMSE* mini-mental state examination, *CMB* cerebral microbleed, *BP* blood pressure, *HbA1c* glycosylated hemoglobin, *LDL-C* low-density lipoprotein cholesterol, *HDL-C* high-density lipoprotein cholesterol. *By Pearson’s chi-square test for categorical variables and one-way analysis of variance test for continuous variables as appropriate. † and ‡ indicate significant difference between them (P < 0.05) by Dunnett’s test for continuous variables or by chi-square test with Bonferroni adjustment for categorical variables (subjects with mild-moderate WMH as the reference group).

### Associations of cSVD phenotypes with vascular endpoints.

During the follow-up period (mean, 1.9 years), stroke was recorded in 117 (8.0%) subjects (93 [6.4%] ischemic stroke, 25 [1.7%] hemorrhagic stroke), MACE in 136 (9.4%) subjects, and all-cause death in 52 (3.6%) subjects. The unadjusted analysis of the association between cSVD phenotypes and outcome events is shown in Table S1. Compared with the reference group, subjects with ICH with advanced WMH had significantly higher risk of ischemic stroke (HR 3.14; 95% CI 1.78–5.55), hemorrhagic stroke (HR 3.18; 95% CI, 1.04–9.73), MACE (HR 2.88; 95% CI, 1.80–4.61), and all-cause death (HR 2.55; 95% CI, 1.15–5.69), while subjects with advanced WMH and subjects with ICH and mild-moderate WMH did not have significantly different risk for these outcomes compared to the reference group.

Tables 2 provides the adjusted HRs for adverse outcomes according to cSVD phenotypes. Compared with the reference group, risk of ischemic stroke was significantly higher in subjects with ICH with advanced WMH (HR 2.62; 95% CI 1.22–5.60). Compared with the reference group, subjects with ICH with advanced WMH were associated with an increased risk of MACE (HR 2.63; 95% CI 1.40–4.95). The risks of hemorrhagic stroke (HR 3.75) and all-cause death (HR 2.86) in subjects with ICH with advanced WMH were numerically higher than that of ischemic stroke, but not significantly different. Fig. S1 depicts the Kaplan–Meier plots for the endpoints of ischemic stroke (A) and MACE (B), respectively among subjects over 2 years by cSVD phenotypes; a higher risk was seen in subjects with ICH with advanced WMH (*P* = 0.0002 and *P* < 0.0001, respectively by log-rank test).

**Table 2:** Adjusted estimates of the HR of cSVD phenotypes for outcome events.

cSVD phenotypes	HR (95% CI)†	*P*	HR (95% CI)*	*P*
	Ischemic stroke		Hemorrhagic stroke	
Mild-moderate WMH	1 [Reference]		1 [Reference]	
Advanced WMH	1.37 (0.69–2.72)	0.371	1.36 (0.36–5.21)	0.651
ICH with mild-moderate WMH	1.16 (0.61–2.23)	0.649	1.07 (0.32–3.59)	0.918
ICH with advanced WMH	2.62 (1.22–5.60)	0.013	3.75 (0.85–16.53)	0.080

	Stroke, MI, or vascular death		All-cause death	
Mild-moderate WMH	1 [Reference]		1 [Reference]	
Advanced WMH	1.20 (0.67–2.15)	0.545	1.45 (0.52–4.07)	0.478
ICH with mild-moderate WMH	1.04 (0.61–1.79)	0.885	1.76 (0.68–4.55)	0.242
ICH with advanced WMH	2.63 (1.40–4.95)	0.003	2.86 (0.91–8.94)	0.071

*cSVD* cerebral small-vessel disease, *WMH* white mater hyperintensity, *MI* myocardial infarction, *HR* hazard ratio, *CI* confidence interval.

*Adjusted for age, sex, smoking, qualifying stroke severity, presence of lacune, presence of CMB, heart rate, mini-mental state examination, glycosylated hemoglobin, triglycerides, uric acid, statin use, antihypertensive use, probucol use, and cilostazol use.

The adjusted HRs of covariates included in the multivariable Cox model appears in Table S2. Among them, probucol medication was found to be an independent predictor of low risk of ischemic stroke and MACE (HR 0.53; 95% CI 0.32‒0.87 and HR 0.65; 95% CI 0.43‒0.97, respectively), while baseline stroke severity, elevated HbA1c, and uric acid levels were linked to an increased risk of MACE (HR 1.09; 95% CI 1.01‒1.19; HR 1.27; 95% CI 1.08‒1.49; and HR 1.19; 95% CI 1.05‒1.36, respectively).

### Subgroup analysis

**Figure 1:**
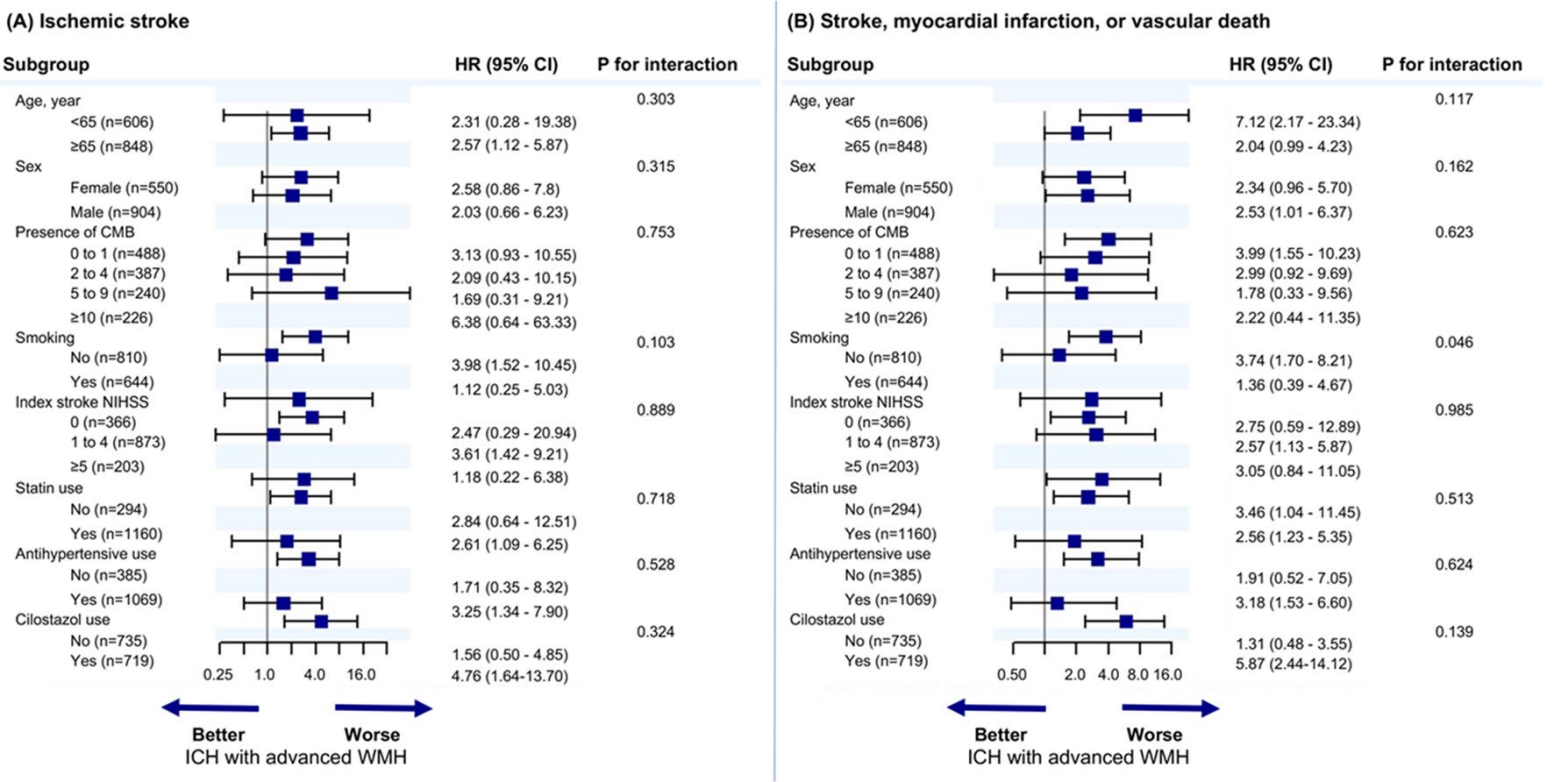
Subgroup analysis of primary and secondary endpoints.

The interaction effects between subgroups and cSVD phenotypes between mild-moderate WMH and ICH with advanced WMH on the risk of key vascular events are shown in Fig. 1. The relative risk of ICH with advanced WMH for the occurrence of (A) ischemic stroke and (B) MACE was consistent (*P*_interaction,_ all > 0.05) across major subgroups stratified by age, sex, CMB severity, smoking, index stroke severity, statin use, antihypertensive use, and cilostazol use.

### Associations of WMH severity with vascular endpoints in subjects with ICH

Baseline demographic and clinical characteristics by WMH severity among 564 ICH-carrying participants are shown in Table 3. Compared with the reference group, subjects with advanced WMH were older, had higher qualifying stroke severity, greater frequencies of presence of lacune, number of CMB, and numerous CMBs (≥ 10). Subjects with advanced WMH were less likely to be male or to be smokers, and MMSE scores, HbA1c and serum levels of uric acid were lower than in the reference group.

**Table 3:** Baseline demographic and clinical characteristics by WMH burden among ICH-carrying participants (N = 564).

	WMH burden			P*
	Mild (n = 192)	Moderate (n = 248)	Advanced (n = 124)	
**Demographics**				
Age, years	60.7 ± 10.7†	66.9 ± 9.9‡	69.0 ± 9.1‡	< 0.001
Male sex	146 (76.0)†	142 (57.3)‡	59 (47.6)‡	< 0.001
**Medical history**				
Hypertension	164 (85.4)†	230 (92.7)‡	116 (93.5)‡	0.014
Diabetes mellitus	68 (35.4)	85 (34.3)	34 (27.4)	0.298
Dyslipidemia	74 (38.5)	97 (39.1)	53 (42.7)	0.733
Coronary heart disease	8 (4.2)	13 (5.2)	4 (3.2)	0.656
Current smoking	103 (53.6)†	96 (38.7)‡	39 (31.5)‡	< 0.001
Family history of stroke	48 (25.0)	53 (21.4)	23 (18.5)	0.382
**Qualifying ischemic event**				0.111
Ischemic stroke	178 (92.7)	240 (96.8)	115 (92.7)	
Transient ischemic attack	14 (7.3)	8 (3.2)	9 (7.3)	
Time to randomization, days	19.5 (8.0, 40.8)	17.0 (6.0, 43.0)	19.5 (12.0, 37.5)	0.619
Qualifying stroke NIHSS	1 (0, 3)	2 (1, 4)	2 (1, 4)	0.093
**MRI marker**				
Presence of lacune	141 (79.2)†	215 (93.5)‡	109 (97.3)‡	< 0.001
CMB, n	2.8 ± 6.8† (n = 178)	5.8 ± 8.8‡ (n = 230)	13.1 ± 21.6‡ (n = 112)	< 0.001
CMB, n 0 to 1 (n = 271)	133 (74.7)†	109 (47.4)‡	29 (25.9)§	< 0.001
2 to 4 (n = 104)	28 (15.7)	49 (21.3)	27 (24.1)	
5 to 9 (n = 70)	12 (6.7)†	39 (17.0)‡	19 (17.0)‡	
≥ 10 (n = 75)	5 (2.8)†	33 (14.3)‡	37 (33.0)§	
**Vital signs**				
Systolic BP, mm Hg	135.4 ± 18.6	134.8 ± 17.5	133.4 ± 16.6	0.616
Diastolic BP, mm Hg	80.5 ± 11.2	80.0 ± 11.2	79.2 ± 10.9	0.574
Heart rate (beats per min)	79.2 ± 13.2	79.4 ± 14.2	81.9 ± 13.0	0.181
Body mass index, kg/m^2^	24.9 ± 3.4†	24.1 ± 3.2‡	23.9 ± 3.5‡	0.024
MMSE, score	25.7 ± 4.3†	22.7 ± 6.0‡	20.1 ± 6.8‡	< 0.001
**Laboratory findings**				
Glucose, mg/dL	123.2 ± 46.5	120.3 ± 43.9	114.1 ± 36.4	0.218
HbA1c, %	6.4 ± 1.3†	6.4 ± 1.4	6.1 ± 1.0‡	0.060
Total cholesterol, mg/dL	169.5 ± 45.1	170.7 ± 42.3	165.4 ± 38.5	0.555
LDL-C, mg/dL	102.3 ± 37.9	103.9 ± 37.5	102.7 ± 35.8	0.912
Triglycerides, mg/dL	134.5 ± 92.3	121.5 ± 73.5	114.8 ± 61.5	0.086
HDL-C, mg/dL	44.0 ± 10.7	45.8 ± 12.3	45.5 ± 12.5	0.297
Creatinine, mg/dL	1.02 ± 0.49	0.97 ± 0.39	0.98 ± 0.67	0.497
Uric acid, mg/dL	5.4 ± 1.8†	4.9 ± 1.6‡	4.8 ± 1.4‡	0.003
**Concomitant medication**				
Antihypertensive	146 (76.0)	177 (71.4)	98 (79.0)	0.239
Statin	150 (78.1)	188 (75.8)	86 (69.4)	0.202
Probucol	89 (46.4)	133 (53.6)	55 (44.4)	0.155
**Antiplatelet drug**				0.431
Cilostazol	97 (50.5)	127 (51.2)	55 (44.4)	
Aspirin	95 (49.5)	121 (48.8)	69 (55.6)	

Values provided are number (%), mean ± SD, or median (interquartile range).

*cSVD* cerebral small-vessel disease, *WMH* white mater hyperintensity, *ICH* intracerebral hemorrhage, *NIHSS* National Institutes of Health Stroke Scale, *MMSE* mini-mental state examination, *CMB* cerebral microbleed, *BP* blood pressure, *HbA1c* glycosylated hemoglobin, *LDL-C* low-density lipoprotein cholesterol, *HDL-C* high-density lipoprotein cholesterol.

*By Pearson’s chi-square test for categorical variables and one-way analysis of variance test for continuous variables as appropriate. † and ‡ or ‡ and § indicates significant difference between them (*P* < 0.05) by Dunnett’s test for continuous variables or by chi-square test with Bonferroni adjustment for categorical variables (subjects with mild-moderate WMH as the reference group).

**Figure 2:**
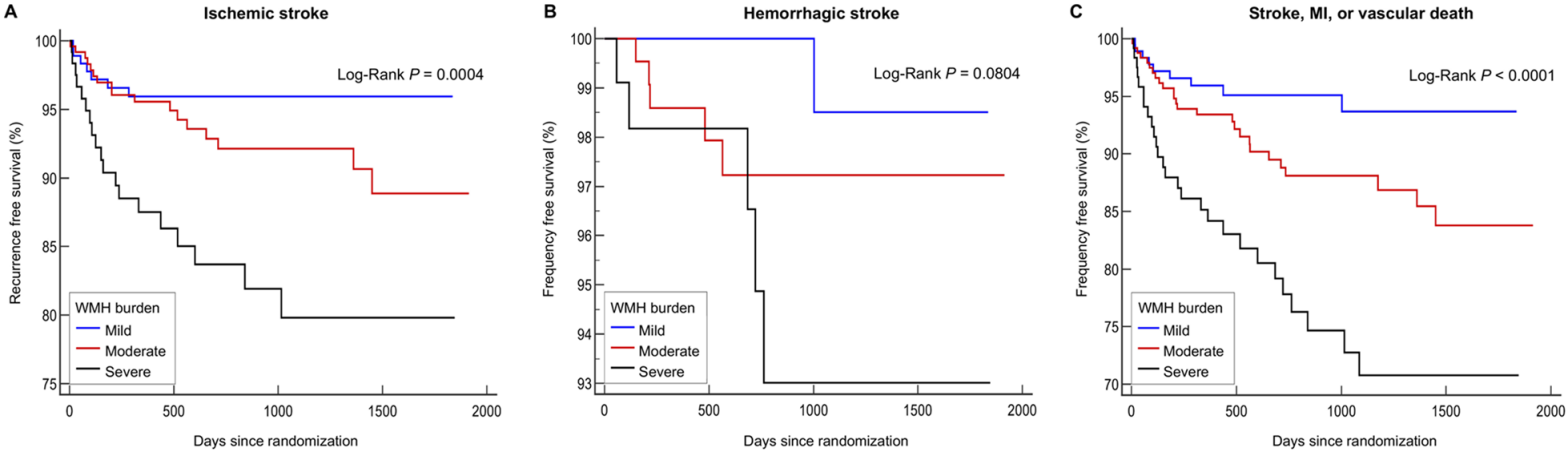
Kaplan-Meier curves for ischemic stroke (**A**), hemorrhagic stroke (**B**), and composite of stroke, myocardial infarction, or vascular death (**C**) among ICH subjects by WMH burden. WMH, white matter hyperintensity.

Tables 4 provides the adjusted HRs for adverse outcomes of WMH with increasing severity among subjects with ICH. Compared with the reference group, the risk of ischemic stroke was numerically higher in subjects with advanced WMH (HR 3.37; 95% CI 0.90‒12.61). Compared with the reference group, subjects with advanced WMH were independently associated with an increased risk of hemorrhagic stroke (HR 33.96; 95% CI 1.52–760.95) and MACE (HR 4.90; 95% CI 1.55–15.54), but not for all-cause death. Figure 2 depicts the Kaplan–Meier plots for the endpoints of ischemic stroke (A), hemorrhagic stroke (B), and MACE (C), respectively among ICH-carrying subjects by WMH burden; a higher risk was noted in ICH subjects with advanced WMH (*P* = 0.0004, *P* = 0.0804, and *P* < 0.0001, respectively by log-rank test).

**Table 4:** Adjusted estimates of the HR for vascular outcomes by ICH-carrying WMH burden.

	Ischemic stroke		Hemorrhagic stroke	
Events, n (%)	43 (7.6%)		11 (2.0%)	
WMH burden	HR (95% CI)*	*P*	HR (95% CI)*	*P*
Mild	1 [Reference]		1 [Reference]	
Moderate	1.73 (0.53–5.61)	0.362	9.03 (0.64–128.32)	0.104
Advanced	3.37 (0.90–12.61)	0.071	33.96 (1.52–760.95)	0.026

	Stroke, MI, or vascular death		All-cause death	
Events, n (%)	62 (11.0)		25 (4.4)	
WMH burden	HR (95% CI)*	*P*	HR (95% CI)*	*P*
Mild	1 [Reference]		1 [Reference]	
Moderate	2.32 (0.83–6.48)	0.107	1.13 (0.20–6.46)	0.887
Advanced	4.90 (1.55–15.54)	0.007	2.10 (0.31–14.16)	0.445

WMH burden	HR (95% CI)*	*P*	HR (95% CI)*	*P*
Mild	1 [Reference]		1 [Reference]	
Moderate	2.32 (0.83–6.48)	0.107	1.13 (0.20–6.46)	0.887
Advanced	4.90 (1.55–15.54)	0.007	2.10 (0.31–14.16)	0.445

*ICH* intracerebral hemorrhage, *WMH* white mater hyperintensity, *MI* myocardial infarction, *HR* hazard ratio, *CI* confidence interval.

*Adjusted for age, sex, hypertension, smoking, qualifying stroke severity, presence of lacune, presence of CMB, body mass index, mini-mental state examination, glycosylated hemoglobin, triglycerides, uric acid, cilostazol use, and probucol use.

## Discussion

In this exploratory study of Asian (primarily Korean) stroke patients at high risk of cerebral hemorrhage, ICH with advanced WMH was significantly associated with an over 2.5-fold increase in risk of recurrent ischemic stroke during the 2-year follow-up period when compared with mild-moderate WMH. Of note, despite the similar settings of higher burden of CMB (> mean 10), which is associated with an increased risk of ischemic stroke^9^, presence of lacune, and similar demographic characteristics with subjects with advanced WMH, the risk of ischemic stroke was greater in subjects with ICH with advanced WMH (HR 2.62 vs. HR 1.37). An independent association was also noted between ICH with advanced WMH and increased risk of MACE. These findings were independent of older age, higher qualifying stroke severity, harboring more lacune and CMBs, cognitive impairment, and low rate of statin medication, and was significant despite a lower frequency of vascular risk factors including male sex and smoking when compared with the reference group. Among high-risk subjects with ICH, the risk of ischemic stroke trended to be higher in subjects with advanced WMH, but Kaplan–Meier curves shown in Fig. 2 showed a significant divergence between mild and advanced WMH in curve for ischemic stroke.

Higher rates of ischemic stroke events in subjects with ICH with advanced WMH despite on secondary stroke prevention in the setting of a prospective and rigorous design is conflicting. The greater burden of cSVD in subjects with ICH with advanced WMH (vs. mild-moderate WMH group or ICH-carrying mild WMH group) might reflect more advanced deep perforating vasculopathy, placing them at relatively higher risk for ischemic events. Another possible explanation might be lower statin medication, poor medication adherence in the setting of older age and impaired cognition, and higher baseline stroke severity^13^.

In this study, the risk of hemorrhagic stroke was numerically higher in subjects with ICH with advanced WMH compared to subjects with mild-moderate WMH (HR 3.75; 95% CI 0.85–16.53). Among individuals with ICH, subjects with advanced WMH were significantly linked to an over 30-fold risk of hemorrhagic stroke, compared with those with mild WMH. These findings are in line with a recent study that 82.6% of acute ICH patients harbored moderate-severe WMH^14^ and highlight the uncertainty surrounding the use of antithrombotic medications^15^. We speculate that the above associations might be potentially mediated by older age, higher frequency of hypertension, greater burden of cSVD including WMH and CMB which reflects more fragile vascular condition, and/or poor medication adherence in the setting of older age and impaired cognition. However, given the total rate of hemorrhagic stroke in patients receiving antiplatelet therapy was 1.7% in the cerebral hemorrhage-prone PICASSO cohort and the event number of hemorrhagic strokes among 564 subjects with ICH was 11 (2.0%), our findings need to be interpreted with caution.

Our findings may be supported by recent observational studies that showed beneficial effects of restarting antithrombotic therapy after ICH on functional outcomes^2,16^ and low risk of all-cause stroke, regardless of hematoma location^2^. Also, the REstart or STop Antithrombotics Randomised Trial (RESTART) provided information on the safety of resuming antiplatelet therapy after ICH, finding fewer recurrences of ICH in patients who resumed antiplatelet therapy when compared to patients who did not resume antiplatelet therapy^17^.

In agreement with our findings, a recent study showed that cumulative incidence rate of ischemic stroke was 3.5-fold higher than hemorrhagic stroke in patients with deep ICH at 5 years of follow-up (11.2% [95% CI 7.2–16.2] vs. 3.2% [1.3–6.3]), while incidence did not strikingly differ in patients with lobar ICH (5.3% [95% CI 2.2–10.6] vs. 7.9% [3.8–13.7])^10^. Our study could not be assessed according to ICH location because ICH location information was not available for review in the PICASSO database. However, subjects in the PICASSO cohort were more likely to have deep ICH, which can be supported from the findings that the risk of ischemic stroke was significantly higher for 2 years and the frequency of hypertension was overwhelmingly higher in subjects with ICH with advanced WMH than in the mild-moderate WMH group. The latter finding is in accordance with the previous study that hypertension is more associated with deep ICH than lobar ICH^18^.

While our main purpose was to investigate the impact of ICH-carrying advanced WMH on the risk of recurrent stroke type, we must point out that despite the lack of significant differences in vascular outcomes between subjects with advanced WMH and ICH subjects with mild-moderate WMH and subjects with mild-moderate WMH, they should not be viewed as low-risk categories. The PICASSO study included patients from a prespecified cSVD cohort, and having a stroke is the strongest predictor of a future stroke^19^, when compared with people who have not had a stroke.

This study has some methodologic limitations. First, this is a post hoc exploratory analysis of a prospective study that evaluated the efficacy and safety of prespecified drugs, and thus our finding that various cSVD phenotypes were associated with certain vascular outcomes do not prove that ICH-carrying advanced WMH are definitely associated with increased risk of adverse vascular outcomes. Second, since this study consisted of Asian (primarily Korean) stroke patients with cSVD recruited from academic centers our findings may not be generalizable to the non-stroke population or other ethnic populations with stroke. Third, the risk of hemorrhagic stroke associated with advanced WMH among ICH-carrying people was excessively greater: the small number of hemorrhagic stroke (n = 11/564) meant that CIs were too wide, jeopardizing the precision of estimates for stroke outcomes. Fourth, information on ICH or CMB location (deep vs. lobar) was not available for review. Furthermore, counting number of CMB from some participating centers was not performed: partial missing data of CMB number might have influenced on multivariable analysis. Last, imaging-based ICH lesion was not distinguished between symptomatic and asymptomatic.

This study has, however several strengths, considering that the PICASSO study was a large MRI-based study of cerebral hemorrhage-prone Asian patients with a high cSVD burden. Also, stroke events were distinguished between ischemic and hemorrhagic types, and risk of vascular events was assessed by comparison with various cSVD phenotypes.

In conclusion, this study demonstrated that among noncardioembolic stroke patients with bleeding-prone conditions, subjects with ICH together with advanced WMH appear to have a higher risk of ischemic stroke, compared with those with mild-moderate WMH, and this risk was higher even than in patients with advanced WMH. Among ICH subjects, WMH burden was associated with increased risk of ischemic stroke by stepwise manner. The risk of hemorrhagic stroke in ICH was more likely higher in accordance with WMH burden. However, given that the event rate was 2.0% among ICH-carrying people and 1.7% among overall PICASSO cohort, hemorrhagic stroke risk might be too small to outweigh the benefits of antiplatelet therapy for secondary prevention^17^. Future large-scale prospective studies warrant to prove the benefit and safety of antithrombotic use among ICH individuals with wider distribution of WMH burden.

## Methods

### Study subjects

The PICASSO (PreventIon of CArdiovascular Events in iSchemic Stroke Patients with High Risk of Cerebral HemOrrhage) trial was a multinational, double-blind, randomized controlled clinical trial conducted across South Korea, Hong Kong, and the Philippines (ClinicalTrials.gov, identified NCT01013532)^20^. The study comprised 1,534 patients who had experienced ischemic stroke or transient ischemic attack within 6 months before enrollment and who had multiple (≥ 2) CMBs or ICH with or without symptoms (≥ 10 mm of hemorrhage on T2*-weighted gradient echo imaging), with a mean follow-up duration of 1.9 years^20^. The main objective of this trial was to evaluate the efficacy and safety of the antiplatelet agent cilostazol with or without probucol (a lipid-lowering cholesteryl ester transport protein activator) in the prevention of cardiovascular events among non-cardioembolic stroke patients at high risk of developing cerebral hemorrhage^20^. The demographic, clinical, laboratory, and brain magnetic resonance imaging (MRI) data were collected at randomization, and subsequent clinical data were obtained during follow-up visits at 1, 4, 7, 10, and 13 months and annually thereafter. Laboratory data were obtained at 1 and 13 months and at the final follow-up visit, and an additional follow-up MRI was conducted after the 13-month visit^20^. Subjects who had no baseline MRI data were excluded from the current analysis.

The study was approved by the Institutional Review Board (IRB) of Asan Medical Center in Seoul, Korea (approval number: 2009-0189 for S.U.K.) and the IRB of Hanyang University Myongji Hospital in Goyang, Korea (approval number: 2009-033 for J.-H.P). Written informed consent was obtained from all participants. All methods were performed in accordance with STROBE guidelines and Declaration of Helsinki.

### cSVD markers

We retrieved information on cSVD markers including WMH, lacune (before qualifying stroke lesion), and CMB. WMH severity and presence of lacune were determined using 1.5 or 3.0 Tesla T2-FLAIR (fluid-attenuated inversion recovery) imaging, and ICH and CMB (< 10 mm in diameter) were identified using T2*-weighted gradient-echo imaging. The severity of WMH was rated, using the visual rating scale proposed by Fazekas scores ranging from 0 to 3^21^. Considering the symmetric distribution of WMH across the midline, WMH was assessed in the hemisphere contralateral to the area affected by acute stroke. WMH of Fazekas score 3 was considered advanced WMH, while Fazecas score 1 and 2 was mild-moderate one. The weighted kappa statistics for the concordance rate for WMH and for the presence of lacune(s) was 0.78 (95% CI; 0.75–0.80) and 0.77 (95% CI; 0.74–0.79), respectively^6^.

### Classification by cSVD phenotypes

**Figure 3:**
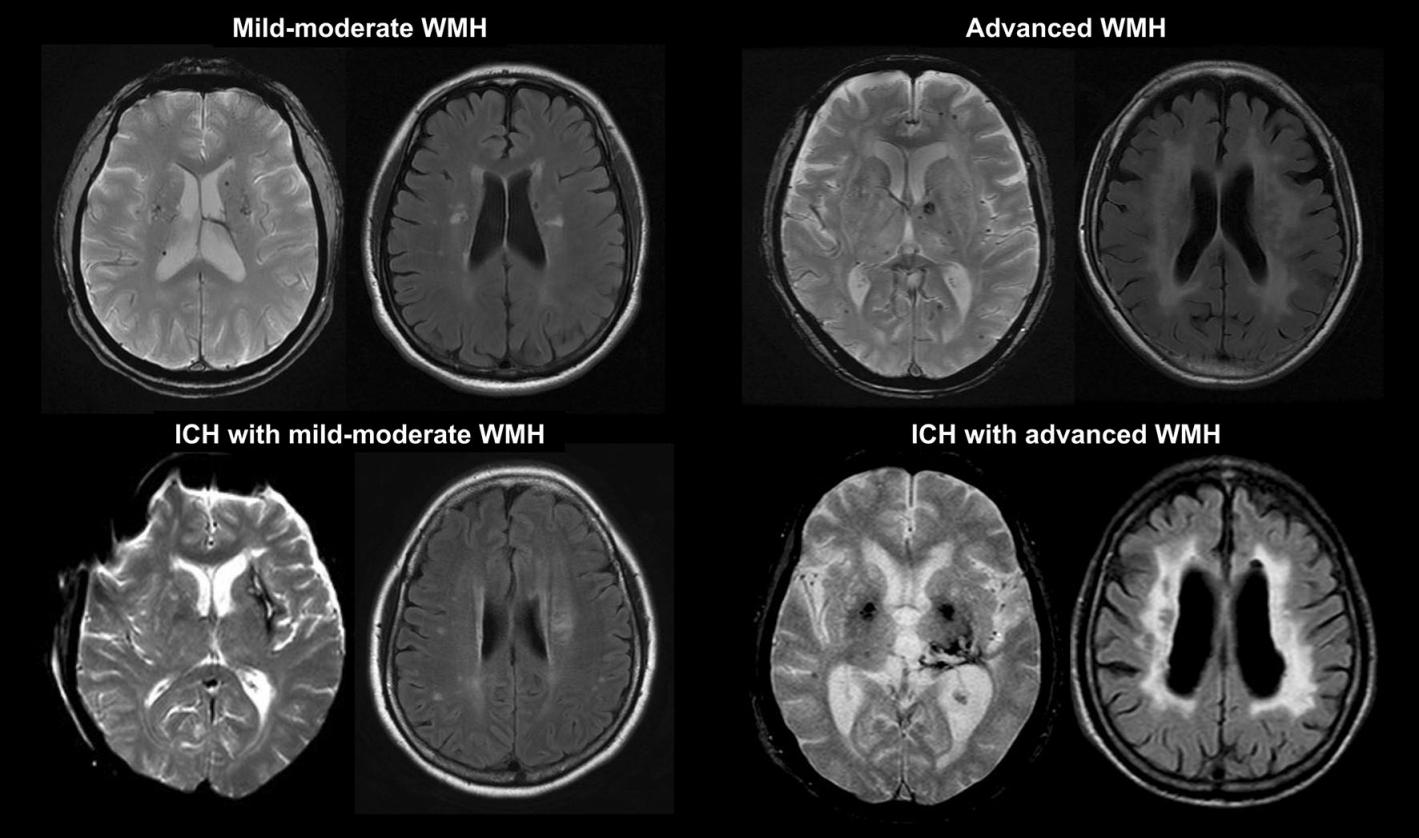
Cerebral small-vessel disease phenotypes.

The PICASSO subjects were categorized into four groups according to the combination of WMH severity with the presence or absence of spontaneous ICH (Fig. 3): subjects with mild-moderate WMH; subjects with advanced WMH; subjects with ICH and mild-moderate WMH; and subjects with ICH and advanced WMH. ICH was defined as imaging evidence, whether symptomatic or not. The reason for setting Fazekas < 3 as the reference class was because of greater associations between WMH by Fazekas score 3 and recurrent stroke risk (ischemic or hemorrhagic)^4,6^. The number of subjects having Fazekas scores of 0, 1, 2, and 3 was 2, 426, 650, and 376, respectively. The two patients with no WMH (Fazekas score 0) were merged into subjects with mild WMH.

### Vascular endpoint assessment

The primary endpoint was ischemic stroke and hemorrhagic stroke, respectively as confirmed by brain MRI or computerized tomography. The secondary endpoint was major adverse cardiovascular events (MACE) including stroke, myocardial infarction, or vascular death. The tertiary endpoint was all-cause death. All vascular endpoints and imaging markers were verified by the Central Independent Adjudication Committee and Imaging Review Committee, respectively, who were blinded to the treatment assignments.

### Statistical analysis

Subjects with mild-moderate WMH were the reference group for the purpose of comparison. Baseline demographic and clinical covariates that influence vascular events after ischemic stroke were preselected based on PICASSO database^20^. Data are presented as mean ± standard deviation (SD), number of subjects (%), or median (interquartile range [IQR]) as appropriate. Comparisons across the groups were examined using Pearson’s chi-square test for categorical variables and the one-way analysis of variance (ANOVA) for continuous variables. Bonferroni’s and Dunnett’s multiple comparisons with the reference group were made for categorical and continuous variables, respectively. Cox proportional hazard regression analyses were performed to estimate the risk of outcome events at 2 years after adjusting for baseline covariates (age, sex, smoking, qualifying stroke severity [National Institutes of Health Stroke Scale], presence of lacune, presence of CMB, heart rate, mini-mental state examination [MMSE], glycosylated hemoglobin [HbA1c], triglycerides, uric acid, and statin use (all *P* < 0.10) and antihypertensive use, probucol use, and cilostazol use. Subjects who did not experience outcome events were censored at the last follow-up examination or the last visit. Subjects lost to follow-up during the study period were included in the Cox proportional hazard model until the last contact. Subgroup comparisons of cSVD phenotypes between mild-moderate WMH ICH and advanced WMH for the hazards of vascular events were performed with respect to subject’s demographics including age (< 65 vs. ≥ 65 years); sex; smoking; index stroke NIHSS (0, 1 to 4, ≥ 5); presence of CMB (0 to 1, 2 to 4, 5 to 9, ≥ 10); and therapeutic medications including statin use, antihypertensive use, and cilostazol use) using Cox proportional-hazards regression model. The subgroup comparisons were made by including appropriate interaction effects between the subgroups and cSVD phenotypes in the multivariable model. The interaction between cSVD phenotypes and variables for predicting the risk of vascular events was assessed by including the appropriate interaction terms in the model. Among ICH subjects, we further assessed the relationships of WMH severity (mild, moderate, and advanced) to predict stroke outcomes after adjusting for baseline covariates (age, sex, hypertension, smoking, qualifying stroke severity, presence of lacune, presence of CMB, body mass index, MMSE, HbA1c, triglycerides, uric acid; all *P* < 0.10), probucol use, and cilostazol use. ICH subjects with mild WMH were set as the reference group. Results were expressed as hazard ratio (HR) and 95% confidence interval (CI). All analyses were conducted using IBM SPSS Version 23.0 (IBM Corp., Armonk, NY). Kaplan–Meier curves were fit by the log-rank tests using MedCalc software version 18.5 (Mariakerke, Belgium) and subgroup analysis was performed using R software (version 3.6.0, r-project.org). A two-sided *P* < 0.05 was considered statistically significant.

## Supplementary Information

Supplementary Information.

## Data Availability

The data used in this study will be made available upon formal request to the corresponding author.
