# Supplementary material for: Prior intracerebral hemorrhage and white matter hyperintensity burden on recurrent stroke risk
Source: Sci Rep. 2021 Aug 31;11:17406. doi: 10.1038/s41598-021-96809-3 (PMC8408204; doi:10.1038/s41598-021-96809-3)
Supplement: Supplementary file 1 — Supplementary Information. [file 41598_2021_96809_MOESM1_ESM.pdf]

Supplementary Material

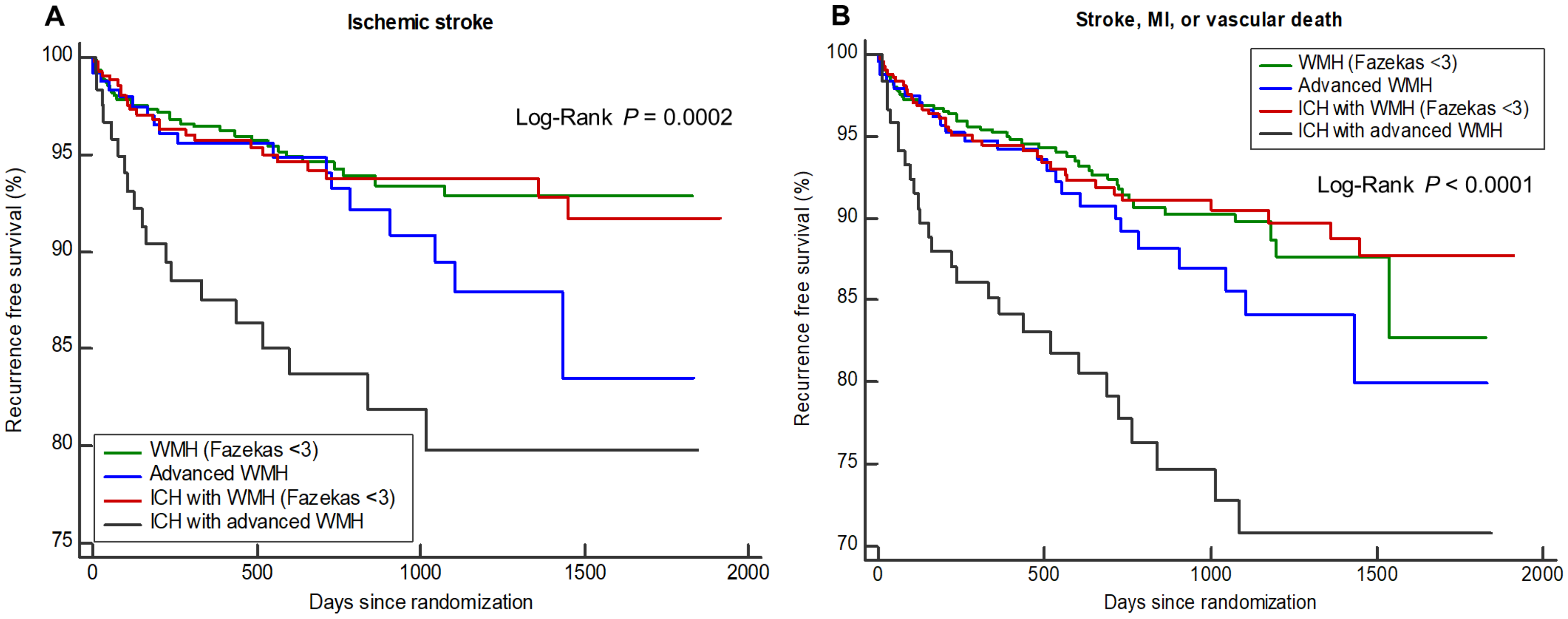

Figure S1. Kaplan-Meier curves for (A) recurrent ischemic stroke and (B) composite of stroke, myocardial infarction, or vascular death by cSVD phenotypes. cSVD, cerebral small-vessel disease.

Table S1. Estimates of the HR of cSVD phenotypes for outcome events (unadjusted)

	cSVD phenotypes			
	Mild-moderate WMH (n=638)	Advanced WMH (n=252)	ICH with mild-moderate WMH (n=440)	ICH with advanced WMH (n=124)
**Ischemic stroke**				
No. of events (incidence rate) /100 person-years	32 (2.66)	18 (4.01)	24 (2.54)	19 (8.08)
HR (95% CI)	1 [Reference]	1.49 (0.83–2.65)	1.04 (0.61–1.77)	3.14 (1.78–5.55)
P		0.179	0.884	<0.001
**Hemorrhagic stroke**				
No. of events (incidence rate) /100 person-years	8 (0.66)	6 (1.34)	6 (0.63)	5 (2.12)
HR (95% CI)	1 [Reference]	2.00 (0.69–5.76)	0.97 (0.34–2.83)	3.18 (1.04–9.73)
P		0.200	0.962	0.043
**Stroke, MI, or vascular death**				
No. of events (incidence rate) /100 person-years	49 (4.08)	25 (5.57)	35 (3.70)	27 (11.48)
HR (95% CI)	1 [Reference]	1.35 (0.83–2.19)	0.97 (0.63–1.50)	2.88 (1.80–4.61)
P		0.222	0.898	<0.001
**All-cause death**				
No. of events (incidence rate) /100 person-years	18 (1.50)	9 (2.00)	16 (1.69)	9 (3.82)
HR (95% CI)	1 [Reference]	1.33 (0.60–2.97)	1.14 (0.58–2.26)	2.55 (1.15–5.69)
P		0.481	0.699	0.022

cSVD cerebral small-vessel disease; *WMH* white mater hyperintensity; *MI* myocardial infarction; *HR* hazard ratio; *CI* confidence interval.

Table S2. AHRs of covariates included in the Cox model (model II) of vascular outcomes by cSVD phenotypes

	Ischemic stroke		Stroke, MI, or vascular death	
Covariates	AHR (95%, CI)	*P*	AHR (95%, CI)	*P*
Age (1-yr difference)	1.02 (1.00–1.05)	0.105	1.02 (1.00–1.04)	0.130
Male sex	1.25 (0.60–2.59)	0.555	0.99 (0.54–1.81)	0.961
Smoking	1.22 (0.65–2.32)	0.537	1.11 (0.64–1.92)	0.706
Qualifying stroke severity	1.09 (0.99–1.21)	0.072	1.09 (1.01–1.19)	0.035
Presence of lacune	2.69 (0.64–11.19)	0.175	2.05 (0.74–5.68)	0.169
Presence of CMB	0.86 (0.46–1.62)	0.645	0.94 (0.55–1.61)	0.823
Heart rate	1.00 (0.98–1.01)	0.645	0.99 (0.98–1.01)	0.336
MMSE	0.96 (0.92–1.01)	0.136	0.98 (0.94–1.02)	0.279
Glycosylated hemoglobin	1.20 (0.99–1.46)	0.070	1.27 (1.08–1.49)	0.003
Triglycerides	1.00 (1.00–1.00)	0.980	1.00 (1.00–1.00)	0.628
Uric acid	1.16 (0.99–1.36)	0.071	1.19 (1.05–1.36)	0.008
Statin use	1.08 (0.59–1.98)	0.798	0.99 (0.60–1.63)	0.980
Antihypertensive use	0.79 (0.47–1.33)	0.375	0.84 (0.54–1.30)	0.426
Probucol use	0.53 (0.32−0.87)	0.012	0.65 (0.43−0.97)	0.037
Cilostazol use	0.86 (0.53−1.39)	0.528	0.92 (0.62−1.39)	0.703

cSVD cerebral small-vessel disease; *CMB* cerebral microbleed; *MMSE* mini-mental state examination; *MI* myocardial infarction; *AHR* adjusted hazard ratio; *CI* confidence interval.
